# Expressional Analysis of Immunoglobulin D in Cattle (*Bos taurus*), a Large Domesticated Ungulate

**DOI:** 10.1371/journal.pone.0044719

**Published:** 2012-09-13

**Authors:** Beilei Xu, Jing Wang, Min Zhang, Ping Wang, Zhiguo Wei, Yi Sun, Qiqing Tao, Liming Ren, Xiaoxiang Hu, Ying Guo, Jing Fei, Lei Zhang, Ning Li, Yaofeng Zhao

**Affiliations:** 1 State Key Laboratory of Agrobiotechnology, College of Biological Sciences, National Engineering Laboratory for Animal Breeding, China Agricultural University, Beijing, P. R. China; 2 College of Animal Science and Technology, Henan University of Science and Technology, Henan, P. R. China; 3 Key Laboratory of Animal Reproduction and Germplasm Enhancement in Universities of Shandong, College of Animal Science and Technology, Qingdao Agricultural University, Qingdao, P. R. China; National Institute on Aging, United States of America

## Abstract

For decades, it has remained unknown whether artiodactyls, such as cattle, pigs, and sheep, express immunoglobulin D (IgD), although the δ gene was identified in these species nearly 10 years ago. By developing a mouse anti-bovine IgD heavy chain monoclonal antibody (13C2), we show that secreted bovine IgD was present mainly as a monomer in serum and was heavily glycosylated by N-linked saccharides. Nonetheless, IgD was detectable in some but not all of the Holstein cattle examined. Membrane-bound IgD was detected in the spleen by western blotting. Flow cytometric analysis demonstrated that IgD-positive B cells constituted a much lower percentage of B cells in the bovine spleen (∼6.8% of total B cells), jejunal Peyer's patches (∼0.8%), and peripheral blood leukocytes (∼1.2%) than in humans and mice. Furthermore, IgD-positive B cells were almost undetectable in bovine bone marrow and ileal Peyer's patches. We also demonstrated that the bovine δ gene can be expressed via class switch recombination. Accordingly, bovine δ germline transcription, which involves an Iδ exon and is highly homologous to Iμ, was confirmed. However, we could not identify an Iδ promoter, despite bovine Eμ demonstrating both enhancer and promoter activity. This study has answered a long-standing question in cattle B cell biology and significantly contributes to our understanding of B cell development in this species.

## Introduction

Immunoglobulin D (IgD) was initially discovered by Rowe and Fahey in the serum of a myeloma patient in 1965 [1]. Since its discovery in humans and mice, IgD has long been thought to exist only in primates and rodents and thus to represent a recently developed Ig class. This belief was not challenged until a milestone paper published by Wilson and colleagues in 1997 that reported the presence of the δ gene in a teleost, the channel catfish (*Ictalurus punctatus*) [2]. Since then, the δ gene has been identified in a large number of species, including nearly all groups of jawed vertebrates (except birds). The discovery of the *Xenopus* δ gene helped clarify that the previously observed IgW in lungfish and sharks also belongs to the IgD lineage [3], [4], [5], [6]. IgD is now believed to be as ancient as IgM in terms of its evolution [3], despite its absence in a number of species, such as birds [7] and select mammalian species, including rabbits and opossums [8], [9].

The δ gene is always located immediately downstream of the μ gene in the immunoglobulin heavy chain (IgH) locus (except perhaps in cartilaginous fish) [10]. This consistency in genomic organization, together with findings from early studies in mice [11], [12], suggests that the δ gene is generally expressed through RNA splicing of a long primary RNA transcript consisting of both μ and δ sequences. That process explains why IgD and IgM can be co-expressed on the B cell surface. The co-transcription pattern differs from class switch recombination (CSR), a pathway utilized in the expression of IgG, IgE and IgA. CSR involves somatic DNA rearrangements that delete the μ and δ genes, as well as other genes upstream of the genes to be expressed. A series of *cis* genetic elements, such as switch regions (S), I exons and I promoters, are involved in the control of CSR [13]. Although rare aberrant IgD switching has been observed in humans and mice [14], [15], [16], it is clear that both the human and mouse δ genes are devoid of a dedicated Sδ region, Iδ exon and Iδ promoters.

In both humans and mice, IgD is primarily expressed in its membrane-bound form (mIgD) and functions as a B cell receptor. IgD is expressed much less frequently in the secreted form (sIgD), although this form is detectable in the serum [17], [18]. Surface IgD is not expressed at the early developmental stages of B cells in primary lymphoid organs, such as bone marrow, where IgM can be expressed. The IgM-positive immature B cells migrate from the bone marrow to peripheral lymphoid organs, where they mature and express surface IgD. In the spleen, IgD-positive cells (either IgM^high^IgD^high^ T2 or IgM^low^IgD^high^ mature B cells) account for a large proportion of conventional B2 B cells [19], [20]. However, IgD is only expressed at low levels in B1 cells, a subset of B cells that constitute a minor fraction of B cells in the spleen and secondary lymphoid tissues but are more frequent in the pleural and peritoneal cavities [21], [22].

Owing to the early failure to detect IgD on the B cell surface or to detect the δ gene in large domesticated animals, such as cows and pigs, these species were initially thought not to express IgD or even to harbor the δ gene [23], [24]. In 2002, however, we clearly demonstrated the presence of the δ gene in these animals [25]. In contrast to their counterparts in humans and mice, the artiodactyl δ gene possesses a CH1 exon that is evidently derived from the μ gene by duplication, as the sequence of δCH1 is nearly identical to the μCH1 sequence in these animals [25], [26]. This event also provides the δ gene with a short Sδ region that is highly homologous to the Sμ. Our preliminary data suggest that the bovine δ gene could be expressed via CSR and the short Sδ could be able to mediate IgD switching in cows. However, it is still unclear whether other *cis* DNA elements, such as the Iδ exon and Iδ promoter, are involved in the process. More essentially, it also remains largely unknown whether the bovine δ gene is expressed at the protein level and how IgD expression is associated with B cell development because of a lack of antibodies directed against bovine IgD. Therefore, in the present study, we performed a thorough analysis of the DNA elements involved in the CSR of the bovine δ gene and developed a monoclonal antibody specific to bovine IgD that can be employed in both Western blot and flow cytometric analyses.

## Materials and Methods

### Ethics statement and animal experimentation

Care of laboratory animals and animal experimentation were performed in accordance with the Beijing Administration Guidelines for the Use of Laboratory Animals. This study and all procedures were approved by the Animal Ethics Committee of China Agricultural University (XK20120501).

### First-strand cDNA synthesis and 3′ RACE PCR

All tissue samples (Holstein cattle) were collected from a local cattle farm (GenProtein Biotech Company Ltd., Beijing, China). Total RNA was extracted from the tissues of one 38-day-old cow using TRIzol (TIANGEN, China) following the manufacturer's instructions. First-strand cDNA was synthesized using approximately 2 µg of total RNA. The specific primers used for 3′ RACE PCR of the δ gene were IgD-3′RACE-GSPA and IgD-3′RACE-GSPB. All primers are displayed in table S1. The resultant PCR products were cloned into the pMD19-T vector (Takara, Dalian) and sequenced.

### RT-PCR and Real-Time RT-PCR

To examine the distribution of IgM, IgD heavy chains and Iμ and Iδ germline transcripts, RT-PCR was performed using the cDNA derived from different tissues. The primer pairs used to amplify the IgM and IgD heavy chain cDNA fragments were cow-μCH2/cow-μCH3 and cow-δCH2/cow-δCH3. The primer pairs used to amplify the bovine Iμ and Iδ germline transcripts were cow-Iμ1/cow-Iμ2 and cow-Iδ1/cow-Iδ2.

To quantify the bovine mIgD and sIgD heavy chain transcripts in different tissues, PCR was conducted using the Applied Biosystems 7900HT Real-Time PCR system (Applied Biosystems, Foster City, CA). Primer pairs, which were designed according to the results of 3′ RACE, were cow-mIgD1/cow-mIgD2, cow-sIgD1/cow-sIgD2 and cow-GAPDH1/cow-GAPDH2. Reactions were run in quadruplicate, and cycle threshold (CT) values were analyzed with SDS software (Applied Biosystems, Foster City, CA) using the comparative CT (ΔΔCT) method.

### PCR amplifications of the recombined Sμ-Sδ DNA fragments

The recombined DNA fragments created by IgM-IgD CSR were amplified from spleen genomic DNA by nested PCR. The primer pairs were Sμ1/Sμ2 and Sδ1/Sδ2. All PCRs were performed with the Phusion® Master Mix with GC Buffer (Finnzymes Oy, Espoo, Finland) following the manufacturer's instructions.

### Enhancer and promoter analysis

A putative Eμ enhancer containing region has been reported in the intron between the bovine JH and IGHM [27]. This region and the sequence located upstream of the Iδ exon were chosen for the enhancer and promoter analysis. These fragments were respectively amplified using the following primer pairs containing *Mlu* I and *Xho* I restriction sites: cow-Eμ-a/cow-Eμ-b, cow-Iδ1-a/cow-Iδ1-b, cow-Iδ2-a/cow-Iδ2-b and cow-Iδ3-a/cow-Iδ3-b. The resulting PCR products, labeled Eμ, Iδ1, Iδ2 and Iδ3, respectively, were cloned into pGL3-promoter luciferase reporter vectors (Promega, Madison, WI). To test for promoter activity, selected fragments were cloned into the pGL3-enhancer and pGL3-basic vectors (Promega). The mouse B cell line X16C8.5 (ATCC, TIB-209) was used in the transfection experiments. The Renilla luciferase-expressing construct pRL-TK (Promega) was used as a transfection efficiency control. The cells were transiently transfected using Lipofectamine 2000 (Invitrogen, Carlsbad, CA) at the dose indicated in the instructions. The transfected cells were cultured for 36 h at 37°C before being harvested and assayed. Luciferase activity was measured using the Dual-Luciferase Reporter Assay System (Promega).

### Preparation and screening of the anti-bovine IgD mAbs

To raise anti-bovine IgD mAbs, the bovine IgD CH2- and CH3-encoding fragments were amplified by primers containing *Nco*I and *Xho*I restriction sites (IgD CH2-3-a and IgD CH2-3-b) and cloned into the prokaryotic expression vector pET-28a(+) (Novagen), in which the fragments were fused to a His-tag for purification by Ni-column chromatography. Using a service from the Abmart company (Shanghai, China), the purified proteins and Freund's adjuvant were used to immunize BALB/c mice. Hybridoma cells were prepared by fusing the splenocytes derived from the immunized mice with Sp2/0 mouse myeloma cells. The specificity of each secreted mAb was examined using ELISA. Selected hybridomas were injected into the mouse peritoneal cavity to induce ascites. The obtained mAbs were further screened by Western blotting on bovine IgD fragments expressed in mammalian cells.

### Western blotting

Splenic membrane proteins were extracted using the Membrane and Cytosol Protein Extraction Kit (Beyotime, China). Serum samples collected from cattle aged 60 days, 180 days, 1 year, 2 years, 3 years, 4 years, 5 years and 6 years were used in the experiment. To examine the glycosylation of IgM and IgD, the serum was treated with the peptides N-glycosidase F (PNGase F, NEB) and endo-α-N-acetylgalactosaminidase (NEB) following the manufacturer's instructions. The primary antibodies used in this study included anti-bovine IgD mAb 13C2 (Abmart, Shanghai, China) and goat anti-bovine IgM polyclonal antibody conjugated to peroxidase (KPL Gaithersburg, MD). The secondary antibody used was peroxidase-conjugated goat anti-mouse polyvalent immunoglobulins (Sigma St. Louis, MO). The target proteins were visualized by an enhanced chemiluminescence (ECL) detection system (Thermo Scientific, Rockford, IL).

### Immunofluorescence

Full-length bovine mIgD heavy chain and FLAG-tagged λ light chain (accession no. BC114801) were cloned into the eukaryotic expression vector pcDNA3.1(+) (Invitrogen). The human embryonic kidney 293T cell line (HEK293T) was purchased from the ATCC (CRL-11268, Manassas, VA). HEK293T cells transfected with plasmids encoding IgD heavy chain and λ light chain or only heavy chain were grown on feeders in 6-well plates for 36 h. One portion was harvested for western blotting, and the other was applied to immunofluorescence to determine whether anti-IgD mAb (13C2) was able to recognize IgD. The secondary antibody used was Alexa Fluor® 594 goat anti-mouse IgG (H+L) (Invitrogen). Stained preparations were examined with an inverted microscope (ECLIPSE Ti-U, Nikon).

### Flow cytometric analysis

The tissue samples were collected from three 1-year-old cows. Leukocytes were isolated from peripheral blood using RBC-lysis buffer (eBioscience, San Diego, CA). Cell suspensions were filtered using 40-µm nylon cell strainers (BD Biosciences, Bedford, MA) before density gradient centrifugation using Ficoll-Paque PLUS (GE Healthcare Biosciences, Sweden). To stain surface marker B220 on bovine B cells, we used mouse anti-bovine CD45R (B220) Clone 10B1652 (USBiological, Swampscott, MA) conjugated to FITC. Sheep anti-bovine IgM-biotin (Bethyl Laboratories, Montgomery, TX) followed by PE/Cy5 Streptavidin (Biolegend, San Diego, CA) were used to label surface IgM on the B cells. Mouse anti-bovine IgD mAb (13C2) conjugated to PE was used to detect the surface IgD marker on the bovine B cells. Staining was performed by following a standard protocol, and the analysis was performed on a MoFlo Flow Cytometer (Beckman).

## Results

### Identification of the 3′ end of the bovine sIgD encoding cDNA

**Figure 1:**
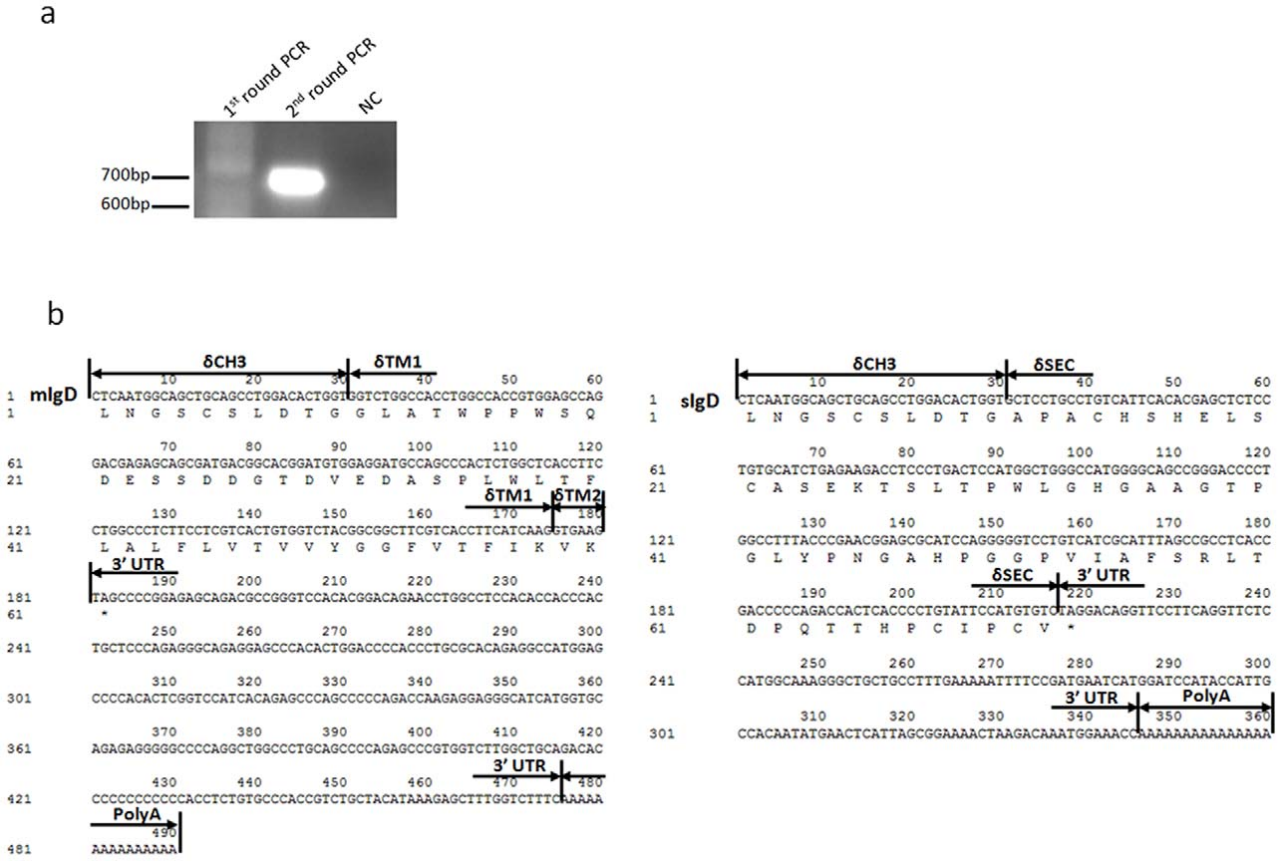
Analysis of the 3′ ends of bovine IgD heavy chain cDNA. (a)PCR product of 3′RACE of the bovine IgD heavy chain cDNA. NC, negative control. (b) The sequence of the 3′ ends of mIgD.

**Figure 2:**
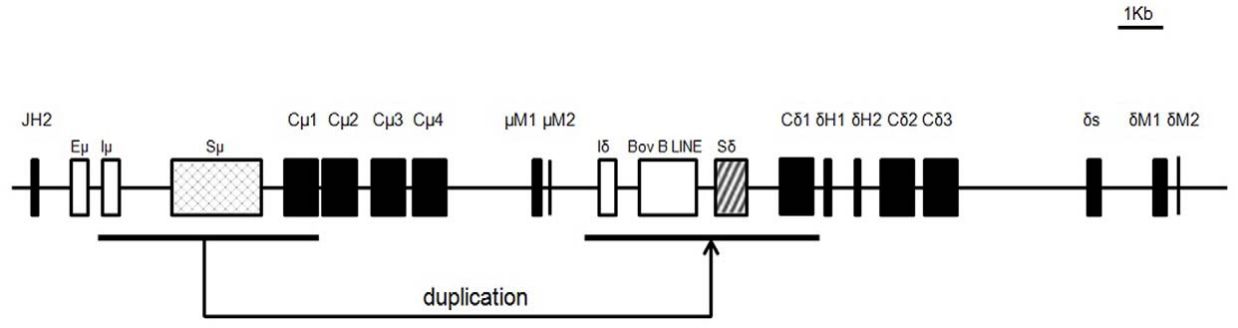
Genomic organization of the bovine Cδ gene. Coding regions are indicated by black boxes. Lower thick lines indicate homologous DNA fragments involved in the duplication. Eμ, 5′ intronic enhancer; Iδ, the putative Iδ exon; Sμ, switch μ; Sδ, switch δ.

While the 3′ membrane-bound tail of the bovine mIgD heavy chain cDNA has already been identified [25], it remains unclear whether bovine IgD can be expressed in a secreted form and, if so, whether the secreted tail is encoded by a separate exon or by a sequence immediately downstream of the terminal CH exon. To address this question, we performed a rapid amplification of cDNA ends (RACE) PCR using primers derived from the bovine δCH3 exon and spleen total RNA as a template. The RACE PCR only generated a major band of ∼700-bp, which corresponds to the expected size of the mIgD heavy chain cDNA tail (**Figs. 1a, 1b**). The PCR products were recovered and cloned. Fifty clones were screened by PCR, which showed that nearly all positive clones contained a 700-bp insert, whereas only one clone had a ∼550-bp insert. Sequencing demonstrated that the 700-bp band was derived from the mIgD heavy chain transcript and that the ∼550-bp band contained a portion of the δCH3 exon and a stretch of sequence distinct from the bovine δTM exon (**Fig. 1b** **, accession numbers: AF515672, JN991202**). This sequence was located in the intron between the δCH3 and δTM1 exons (**Fig. 2**), and it apparently represented a separate exon encoding 62 amino acids. We were able to amplify the IgD heavy chain transcript spanning VDJ-Cδ attached at the 3′ end by this exon, suggesting that similar to humans and mice, cattle also use a single dedicated exon to encode the IgD secreted tail.

### The bovine δ gene is primarily expressed in the spleen and peripheral blood leukocytes

**Figure 3:**
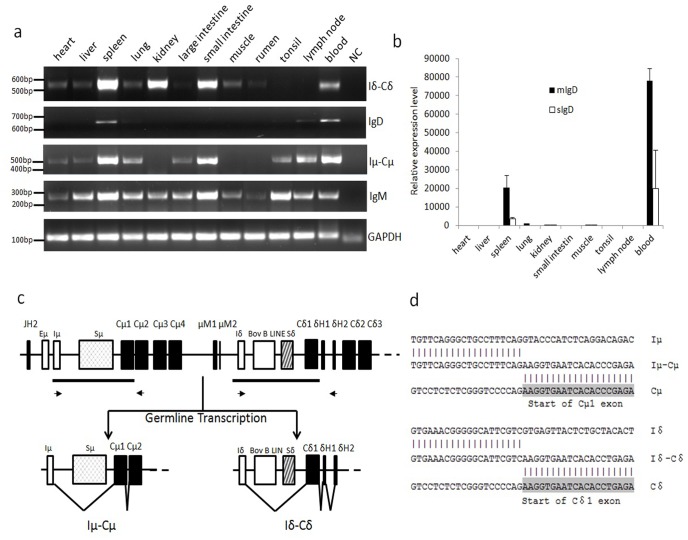
Transcription of the bovine μ and δ genes in different tissues. (a) RT-PCR detection of the IgD, Iδ germline, IgM and Iμ germline transcripts in different tissues. GAPDH was used as an internal control. (b) Q-PCR analysis of mIgD and sIgD in different tissues. Data were analyzed using the ΔΔCT method. GAPDH was used as an internal control. (c) Germline Iμ-Cμ and Iδ-Cδ transcripts. Lower thick lines indicate homologous DNA fragments, whereas arrows indicate primers for Iμ and Iδ germline transcripts. (d) Sequence of Iμ-Cμ and Iδ-Cδ transcripts from the spleen.

Based on the sequences of sIgD obtained above and the mIgD heavy chain transcripts, we designed primers that distinguished the mIgD and sIgD transcripts and then detected their respective expression in different tissues using quantitative PCR. The data show that bovine IgD was mainly expressed as mIgD transcripts in the spleen and peripheral blood leukocytes (PBLs) (**Figs. 3a, 3b**).

### Germline transcription of the bovine δ gene

Germline (GL) transcription of immunoglobulin heavy chain constant (IGHC) genes, which is driven by I rather than VH promoters [13], is thought to be involved in the initiation of CSR. I promoters are typically located immediately upstream of I exons, 5′ to the S regions of each IGHC gene. Thus, GL transcripts are characterized by the splicing of an I exon onto each IGHC gene instead of a rearranged VDJ exon. Because the bovine δ gene can be expressed through CSR, we determined whether there were germline δ transcripts. We first performed a BLAST search against the bovine EST database deposited in the NCBI GenBank using the intron sequence between the μ and δ genes. This search identified an EST clone (accession no. DY150134) containing a spliced product of a short sequence of the δCH1 or μCH1 exon located approximately 1.3 kb downstream of the μTM1 and 3.6 kb upstream of the δCH1 (**Fig. 3c**). It was difficult to judge whether this EST was expressed from δ or μ as the bovine μCH1 and δCH1 are similar in sequence, and the spliced sequence stretch can also be observed in the JH-Cμ intron (approximately 0.4 kb downstream of the putative Eμ enhancer and 1 kb 5′ to the Sμ). We thus designed a sense primer located in the spliced sequence and two anti-sense primers, one in μCH2 and another in the δ hinge exon (**Fig. 3c**). These two anti-sense primers would distinguish the δ transcripts from the μ transcripts. In combination with the sense primer, both anti-sense primers generated specific bands. Sequencing data confirmed that the amplified bands were either the sequence spliced to the μ or δ (**Fig. 3d**), suggesting that the bovine μ and δ genes utilized a homologous sequence stretch as their respective Iμ and Iδ exons.

Using the abovementioned primers, we detected both the μ and δ GL transcripts in different tissues (**Fig. 3a**). The δ GL transcript was mainly detected in the spleen, small intestine, kidney and blood.

### The bovine δ gene can be expressed by CSR

**Figure 4:**
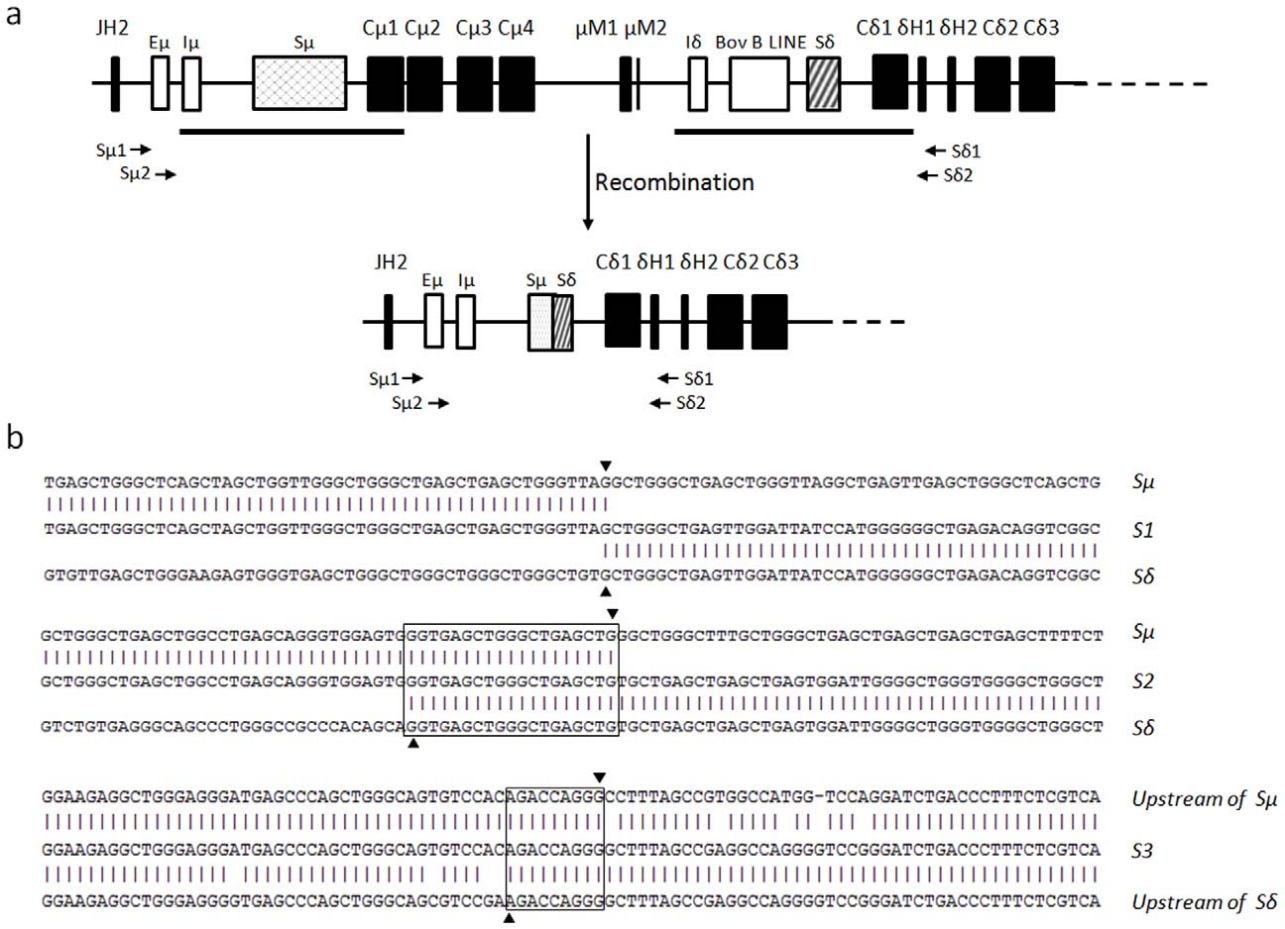
Switch recombination between Sμ and Sδ. (a) The schematic map of the CSR of the Cδ gene. Arrows indicate the positions of the Sμ1, Sμ2, Sδ1 and Sδ2 primers. (b) DNA sequence of the recombined Sμ-Sδ junctions. Upper sequence, bovine germline Sμ region (accession no. AY158087); lower sequence, bovine germline Sδ region (accession no. AF411241). The middle sequences are the cloned PCR products. Identical nucleotides are shown by vertical lines. The Sμ and Sδ breakpoints are represented by an inverted triangle and forward triangle, respectively. Overlaps are indicated by boxes.

The δ gene in humans and mice is typically expressed through co-transcription with the μ gene, and alternative RNA splicing of a long primary transcript generates either IgM or IgD heavy chain transcripts [11], [12]. In rare cases, the δ gene can be expressed through CSR, whereby the μ gene is deleted and the δ gene placed immediately downstream of the rearranged VDJ exon [28]. In our previous studies, preliminary data generated by the amplification of Sμ-Sδ junctions demonstrated that the bovine δ gene could be expressed via CSR [25]. One concern was that the primers used in that study were derived from the Sμ and Sδ flanking regions, which were highly homologous to one another because of a DNA duplication event [25]. This situation may generate problems when analyzing the Sμ-Sδ junctions. To resolve this issue, we redesigned two pairs of primers located outside of the homologous regions in this study (**Fig. 4a**). In combination with long-distance PCR and using spleen genomic DNA as a template, these primers were used in a two-round nested PCR, which generated products only if DNA recombination between the Sμ and Sδ had occurred. Using this approach, PCR fragments ranging from 3-kb to 5-kb in size were generated and subsequently sequenced. We then identified the recombination breakpoints by comparing the obtained sequences with the germline Sμ and Sδ (**Fig. 4b** **, S1**). The identified breakpoints were located either within or outside of the S regions. Very few Sμ-Sδ junctions demonstrated direct end joining of the Sμ and Sδ. Most junctions displayed a stretch of sequence shared by Sμ and Sδ, suggesting a preference for using the microhomology-based end-joining pathway [29]. These data clearly demonstrate that the bovine δ gene could be expressed through a CSR process.

### Eμ, but not the sequence upstream of Iδ, shows enhancer and promoter activities in mouse B cells

**Figure 5:**
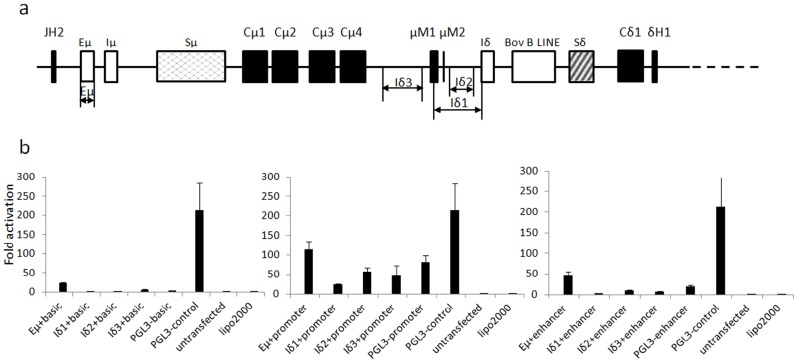
Promoter activity analysis of the sequences upstream of the Iδ exon. (a) Schematic positions of the cloned fragments tested for promoter activity. Eμ has been previously described [27]. Eμ, Iδ1, Iδ2 and Iδ3 indicate the cloned fragments. (b) Detection of the promoter or enhancer activity in X16C8.5 B cell lines. Eμ, Iδ1, Iδ2 and Iδ3 were separately cloned into the pGL3-basic, pGL3-promoter and pGL3-enhancer vectors and named Eμ+basic, Eμ+promoter and Eμ+enhancer, respectively. pGL3-control was a positive transfection control. pGL3-basic, pGL3-promoter, pGL3-enhancer and untransfected cells were used as the controls. The average values of three replicates and standard deviations are shown.

In humans and mice, GL transcription of γ, ε and α genes is driven by their respective I promoters, whereas the μ GL transcription is initiated by Eμ [30]. Detectable GL transcripts of δ gene in cattle indicate that a dedicated Iδ promoter might also be present immediately upstream of the Iδ exon. We cloned three fragments spanning the region from μCH4 to Iδ in addition to the previously predicted Eμ enhancer, into a series of luciferase reporter vectors (**Fig. 5a**). The resultant constructs were used to transfect a mouse B cell line (X16C8.5). As shown in **Fig. 5b**, bovine Eμ demonstrated both enhancer and promoter activities in the transfected cells, while neither promoter nor enhancer activity was detected for the sequence upstream of the Iδ exon in the transfected mouse B cells. However, we can not absolutely exclude the possibility of the presence of a bovine Iδ promoter only based this transfection experiment, as the putative Iδ promoter may not be functional in mouse B cells. Additionally, if the putative bovine Iδ promoter is not a constitutive promoter, its activities may only be detectable under specific conditions.

### Development of a mouse anti-bovine IgD mAb 13C2

Sixteen hybridoma clones were obtained from Abmart (Shanghai, China). Supernatants from three hybridomas produced strong signals of an expected size when performing a western blot analysis using lysates of HEK293T cells transfected with a eukaryotic expression vector pcDNA 3.1(+), in which the bovine δCH2 and δCH3 portions were cloned (data not shown). However, only one clone (13C2) detected a single specific band when used in western blots of bovine serum. We made two additional eukaryotic expression constructs containing either the δCH2 or δCH3 gene and transfected them into HEK293T cells separately. The subsequent Western blot suggested that the 13C2 mAb detected a sequence in the δCH3 domain (data not shown).

**Figure 6:**
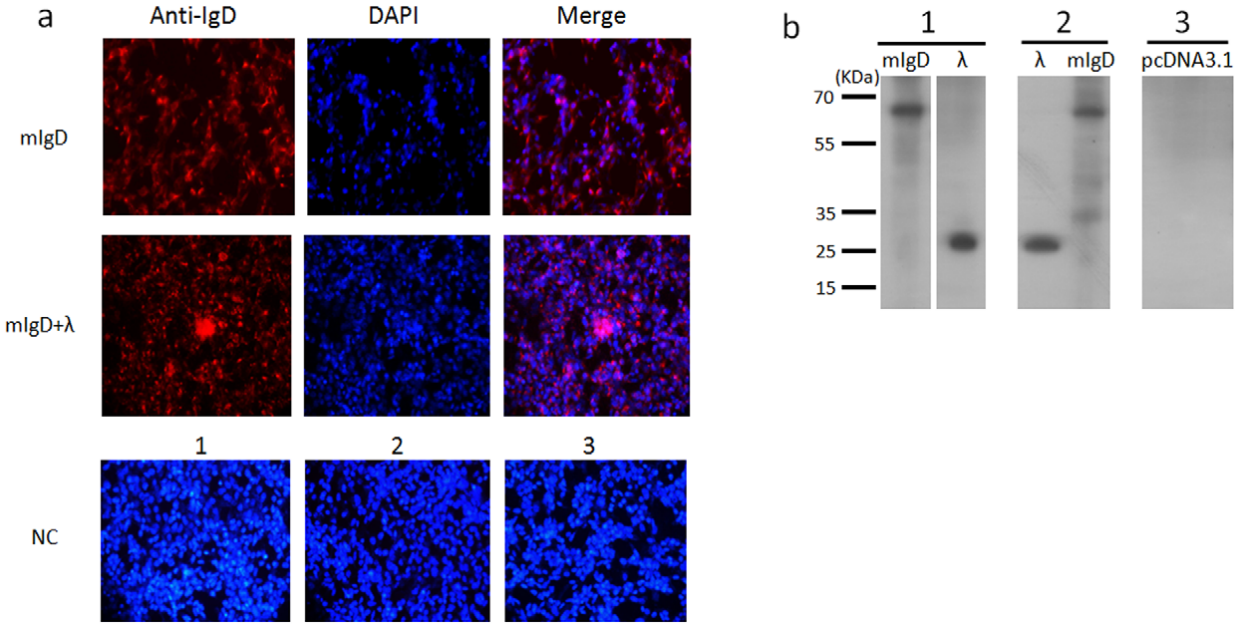
Immunofluorescence staining using the 13C2 mAb. (a) Immunofluorescence staining of full-length mIgD heavy chain or λ light chain expressed on HEK293T cells with mAb 13C2(anti-IgD) plus goat anti-mouse IgG (red) and DAPI (blue). mIgD, cells transfected with full-length mIgD plasmid only; mIgD+λ, cells transfected with full-length mIgD heavy chain and λ light chain plasmids together. NC, negative control. 1, Cells transfected with an mIgD plasmid, stained by the secondary antibody goat anti-mouse IgG. 2, Cells transfected with mIgD and λ plasmids, stained by the goat anti-mouse IgG. 3, Cells transfected with the empty vector pcDNA3.1(+), stained by 13C2 plus goat anti-mouse IgG. Original magnification, ×20. (b) Immunoblot analysis of bovine mIgD heavy and light chains in cell lysates. mAb 13C2 was used to detect mIgD, and anti-FLAG antibody was used to detect the λ light chain. 1, Cell lysates from HEK293T cells transfected with mIgD heavy chain and λ light chain plasmids separately. 2, Cell membrane proteins extracted from HEK293T cells transfected with mIgD and λ plasmids together. 3, Negative control of membrane proteins from HEK293T cells transfected with empty vector pcDNA3.1(+).

The above experiments suggested that the 13C2 mAb could be used in western blot detection of bovine IgD. We next examined whether this mAb could be used in immunofluorescence. Two constructs containing the full-length mIgD heavy chain or the bovine λ light chain cDNA tagged with FLAG were transfected into HEK293T cells either separately or together. Immunostaining with 13C2 revealed that the cells transfected with the mIgD construct or both the mIgD and light chain constructs were stained (**Fig. 6a**). Western blotting using anti-FLAG antibodies produced clear bands of ∼26 kDa for λ chain and ∼65 kDa for the mIgD heavy chain when using mAb 13C2 (**Fig. 6b**).

The mIgD heavy and light chain double-transfected cells were also subjected to flow cytometric analysis using PE-labeled 13C2. The results confirmed that the mAb can also be used in flow cytometric analysis (data not shown).

### Detection of secreted and membrane-bound endogenous bovine IgD by Western blotting

**Figure 7:**
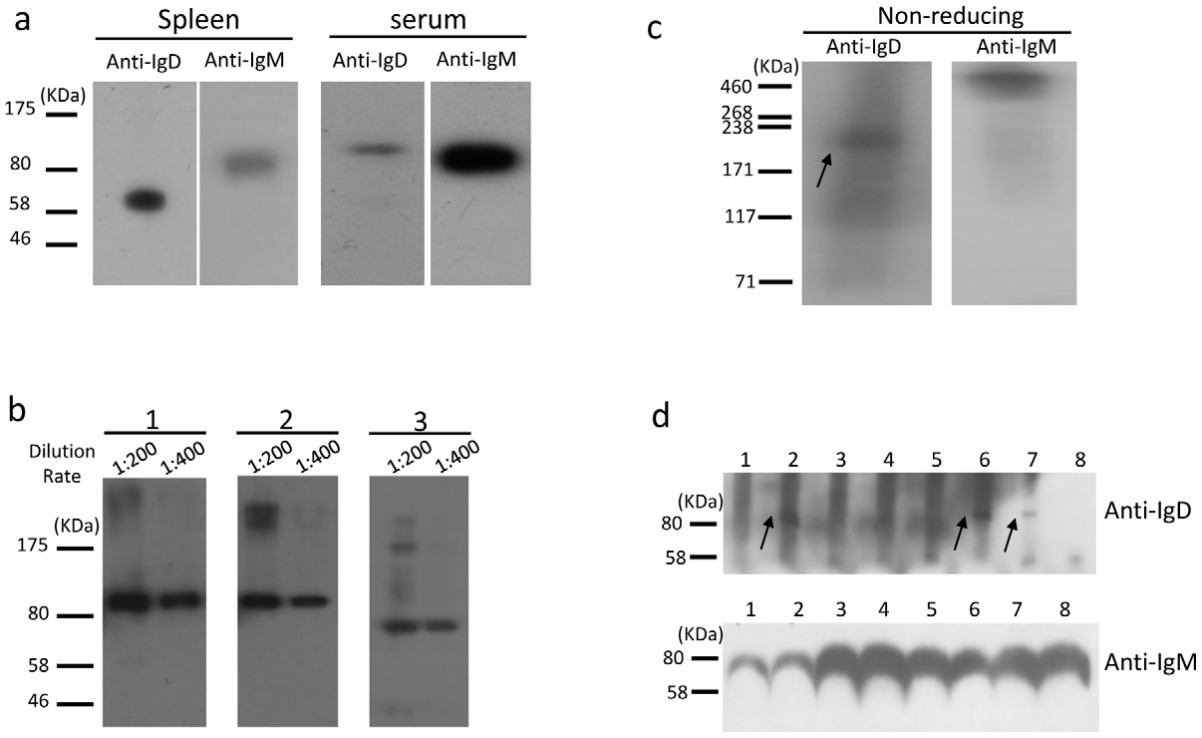
Immunoblot analysis of endogenous bovine IgD. (a) Western blot of splenic membrane and serum proteins. (b) Western blot analysis of the IgD glycosylation in bovine serum. 1, Untreated serum proteins; 2, endo-α-N-acetylgalactosaminidase-treated serum proteins; 3, PNGase-treated serum proteins. The primary antibody 13C2 was diluted at 1∶200 or 1∶400. (c) Serum immunoblot analysis with mAb 13C2 and anti-bovine IgM polyclonal antibody under nonreducing conditions. The arrow indicates the monomer of IgD. (d) Immunoblot detection of serum IgD and IgM in differently aged cows. 1, 60 days old; 2, 180 days; 3, 1 year; 4, 2 years; 5, 3 years; 6, 4 years; 7, 5 years; 8, 6 years. The arrows indicate the IgD heavy chain.

Because the PCR data suggested that bovine IgD was mainly expressed in the spleen and PBLs, both the splenic membrane and serum proteins were prepared and used for western blotting using 13C2. Under reducing conditions, an obvious band of ∼65 kDa was detected in the splenic membrane proteins (**Fig. 7a**), which agrees closely with the predicted molecular weight of mIgD. A weak but specific band of >80 kDa was detected in serum (**Fig. 7a**), which was approximately 10–20 kDa larger than the predicted size, suggesting a heavy glycosylation of sIgD. To address the latter issue, the serum proteins were treated with either PNGase F (which cleaves N-linked saccharides) or endo-α-N-acetylgalactosaminidase (which cleaves O-linked saccharides) prior to performing the Western blot. A smaller band was observed in PNGase F-treated serum, whereas the size of the detected band was either unchanged or only slightly changed when treated by endo-α-N-acetylgalactosaminidase, indicating that bovine sIgD in serum mainly contains N-linked glycosylations (**Fig. 7b**).

Under non-reducing conditions, mAb 13C2 detected a predominant band with a molecular mass of ∼210 kDa in serum (**Fig. 7c**), whereas a band with a molecular mass of >460 kDa was detected with an anti-bovine IgM polyclonal antibody (**Fig. 7c**). This finding indicates that the bovine sIgD was present in serum mainly in a monomeric form (two >80 kDa IgD heavy chains plus two light chains), whereas sIgM was either dimeric or polymeric.

To detect IgD expression in different animals, serum samples collected from 8 Holstein cows aged 60 days to 6 years were used for western blotting. sIgD was only detectable in three cows (aged 6 months, 4 years and 5 years), suggesting a variable expression (**Fig. 7d**). To exclude the possibility that the variable expression was due to allotypic differences, we sequenced the δCH3 exon and found no sequence variations in these animals.

### Flow cytometric analysis of bovine B cell populations using 13C2

**Figure 8:**
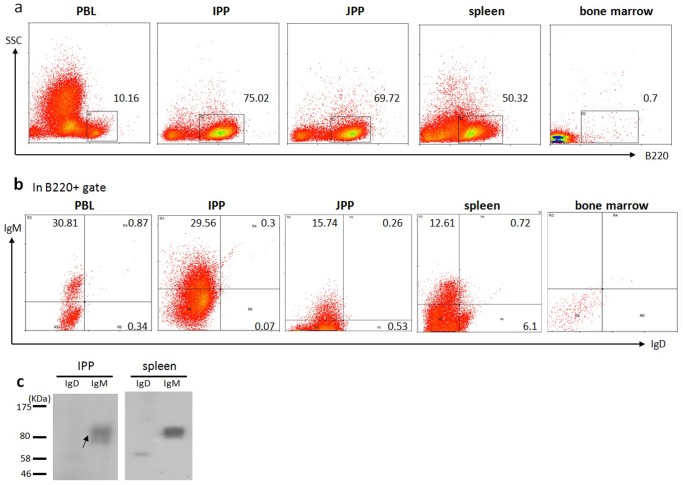
Flow cytometric analysis of B cell populations in the PBLs, ileum PP, jejunum PP, spleen and bone marrow from a 1- year-old cow. (a) Cells were stained for B220, and the proportion of cell populations are indicated for each gate. (b) FACS analysis of lymphocytes from the PBLs, IPP, JPP, spleen and bone marrow using FITC-conjugated anti-bovine B220, PE-conjugated anti-bovine IgD (13C2) and biotin-labeled anti-bovine IgM plus PE/Cy5-labeled streptavidin. Numbers adjacent to the outlined areas indicate the percent of IgM+ cells or IgD cells in the B220 gate. (c) Western blotting of membrane proteins extracted from the ileal PP and spleen of the same cow using anti-bovine IgD and anti-bovine IgM separately. Note: three animals were used in this experiment, but only representative data from one animal are shown.

Using anti-B220, anti-bovine IgM and 13C2 (anti-bovine IgD) antibodies, we performed flow cytometric analysis of bovine B cells derived from a number of different tissues of three one-year-old cows, including the bone marrow, spleen, PBLs, IPP and JPP. Similar results were obtained regarding the percentage of IgD-positive B cells in different tissues from all three cows. Representative data from one animal are shown in Fig. 8. Whereas few B220-positive cells were detected in the bone marrow, relatively larger proportions of B220-positive cells were observed in the PBLs, spleen IPP and JPP (**Fig. 8a**). IgD-positive B cells were rarely detected in the IPP and only in slightly higher numbers in the JPP (∼0.8%) and PBLs (∼1.2%), whereas they were relatively more frequent in the spleen (∼6.8%) (**Fig. 8b** **, S2**). This finding is consistent with the Western blot results in tissues from the same animal, which revealed IgD to be detectable in the spleen, whereas little or no IgD was found in the other tissues **(** **Fig. 8c**). The proportion of IgD-positive cells was significantly lower than that of IgM-positive B cells in all tissues examined.

## Discussion

In the present study, we performed a thorough expression analysis of the immunoglobulin δ gene in a large domesticated animal. We revealed that the bovine δ gene can be expressed via a CSR process. With the successful development of a mouse anti-bovine IgD monoclonal antibody (13C2), we demonstrated bovine IgD expression at the protein level in serum and on the B cell surface for the first time.

CSR is typically involved in the expression of the immunoglobulin heavy chain γ, ε and α genes located downstream of the μ and δ genes, requiring deletions of large DNA regions between the Sμ and a downstream S region of the gene to be expressed. In humans and mice, the δ gene is typically expressed by co-transcription with the μ gene and is rarely expressed through CSR. Neither a dedicated Sδ nor Iδ promoter is found in these species. In artiodactyls, such as cows and pigs, we previously found that a DNA duplication of the μCH1 exon and its 5′ flanking sequence into the δ gene created a short Sδ region that is highly homologous to Sμ. We have now demonstrated that Sδ can mediate IgD CSR and that there is an Iδ exon in cows.

Although not directly demonstrated in this study, the finding of CSR between the bovine μ and δ genes strongly suggests the presence of IgM-IgD+ B cells that can make IgD antibodies and suggests that IgD may be involved in the immune response against pathogens in cattle. Cerutti and colleagues recently found abundant IgM-IgD+ B cells in human upper respiratory mucosa and that secreted IgD antibodies could recognize respiratory bacteria [28], implying a role for IgD in mucosal immunity. These IgM-IgD+ B cells may also be transmitted from the respiratory mucosa to mammary, salivary and lacrimal sites and enhance immune protection against local pathogens and commensal bacteria. In this regard, IgM to IgD switching may be a result of antigen exposure, and IgM-IgD+ B cells may have undergone affinity maturation. In addition to playing a role in mucosal immunity, the secreted IgD may also be involved in systemic immunity through interacting with circulating innate effector cells (basophils) [28]. As this study clearly showed that the bovine IgD can be expressed through CSR and detected in the serum, it would be interesting to investigate whether it performs similar functions in cattle.

According to PCR, western blotting and flow cytometry results using the bovine IgD-specific mAb that we developed, bovine IgD is mainly expressed in the membrane-bound form in the spleen. As in humans and mice, the secreted form of bovine IgD is expressed only at a low level, as we were able to detect sIgD in the serum of only some individuals. Western blotting also revealed bovine sIgD to be present in the serum mainly as a monomer, and the heavy chain was heavily glycosylated by N-linked saccharides. Nearly no O-linked saccharides of bovine sIgD were detected. This situation differs from human IgD, which is glycosylated by both N- and O-linked saccharides [31], [32]. In humans, all types of Ig heavy chains are modified by N-linked saccharides, while only IgD and IgA contain O-linked saccharides, which are used to bind IgD receptors in human T cells [33]. Glycosylations are tightly associated with the biological functions of secreted antibodies, as changes in the glycosylation pattern could cause the antibody function to shift between pro-inflammatory and anti-inflammatory [34]. It is expected that the heavy N-linked glycans in bovine IgD are important for its immunological roles.

In contrast to rodents and humans, in which B cells are generated in the bone marrow throughout life, for most domesticated species, such as rabbits, sheep and cattle, it is uncertain where B lymphopoiesis takes place in adult animals [35], [36], [37], [38], [39], [40]. In this study, a limited number of B cells were detected in the bone marrow of a 1-year-old cow compared with the spleen, ileum and jejunum (**Fig. 8**). There are two types of Peyer's patches in cattle: the IPP, arguably the primary lymphoid organ of B cell development; and the JPP, which plays a role as a secondary lymphoid tissue [41]. Similarly, almost no IgD-positive B cells were detected in the IPP. The IgD-positive B cells were relatively enriched in the spleen (∼6.8%). Thus, it appears that bovine IgD tends to be expressed in B cells at more mature developmental stages, similar to what is observed in humans and mice. However, the proportion of B cells that are IgD-positive is lower in cows than in humans and mice (>70% of B cells in the mouse spleen; >60% of B cells in human PBLs) [28], [42], [43]. IgM and IgD double-positive B cells also constitute a minor proportion of B220-positive cells in all examined tissues of cows (∼0.87% in the PBLs and ∼0.72% in the spleen), which may explain why previous attempts using immunoprecipitation with anti-bovine light chain antibodies to identify mIgD worked in humans and mice but failed in cows [24].

Although its biological significance is unclear, the low proportion of IgD-positive B cells in cattle is another striking difference regarding the biology of lymphocytes and their receptors (BCR and TCR) compared with humans and mice. Several distinct features have been defined for bovine immunoglobulin heavy chains, such as the use of a single VH family in their repertoire and unusually long CDR3 (20 codons on average) [44], [45], [46], [47], [48], [49]. The long CDR3 is primarily attributed to the long germline DH involved in VDJ recombination; the longest DH is 148 bp in size, encoding 49 amino acids [50]. When infected by certain pathogens, cattle tend to respond with a proliferation of IgM^+^CD5^+^ B cell populations [51], which produce low-affinity but polyreactive IgM with activity toward autoantigens. Furthermore, a naturally more innate population of γδ T cells is also found at a significantly higher proportion in cattle than in humans or mice [52], [53]. It would be interesting to address the immunological significance and underlying mechanisms of these species differences in the immune system.

In summary, we present a detailed characterization of IgD expression in cattle and report bovine IgD expression at the protein level for the first time. The successful development of a monoclonal antibody against bovine IgD would significantly aid our understanding of the development of B cells in cattle.

## Supporting Information

Table S1 Primers used in this study.
(TIF)

Figure S1 **DNA sequence of the recombined Sμ-Sδ junctions.** Upper sequence, bovine germline Sμ region (accession no. AY158087); lower sequence, bovine germline Sδ region (accession no. AF411241). The middle sequences are the cloned PCR products. Identical nucleotides are shown by vertical lines. The Sμ and Sδ breakpoints are represented by inverted triangles and forward triangles, respectively. Overlaps are indicated by boxes.
(TIF)

Figure S2 **Control plots for the flow cytometric analysis.** FACS dot plots show the fluorescence of unstained cells from the PBLs, IPP, JPP, spleen and bone marrow separately (top panel) and the fluorescence area of cells stained with either PE-conjugated anti-bovine IgD (second panel) or biotin-labeled anti-bovine IgM plus PE/Cy5-labeled streptavidin (third panel) alone. The bottom panel shows the result of FACS using FITC-conjugated anti-bovine B220, PE-conjugated anti-bovine IgD and biotin-labeled anti-bovine IgM plus PE/Cy5-labeled streptavidin together.
(TIF)
