# Supplementary figures and images for: Expressional Analysis of Immunoglobulin D in Cattle (Bos taurus), a Large Domesticated Ungulate
Source: PLoS One. 2012 Sep 13;7(9):e44719. doi: 10.1371/journal.pone.0044719 (PMC3441446; doi:10.1371/journal.pone.0044719)

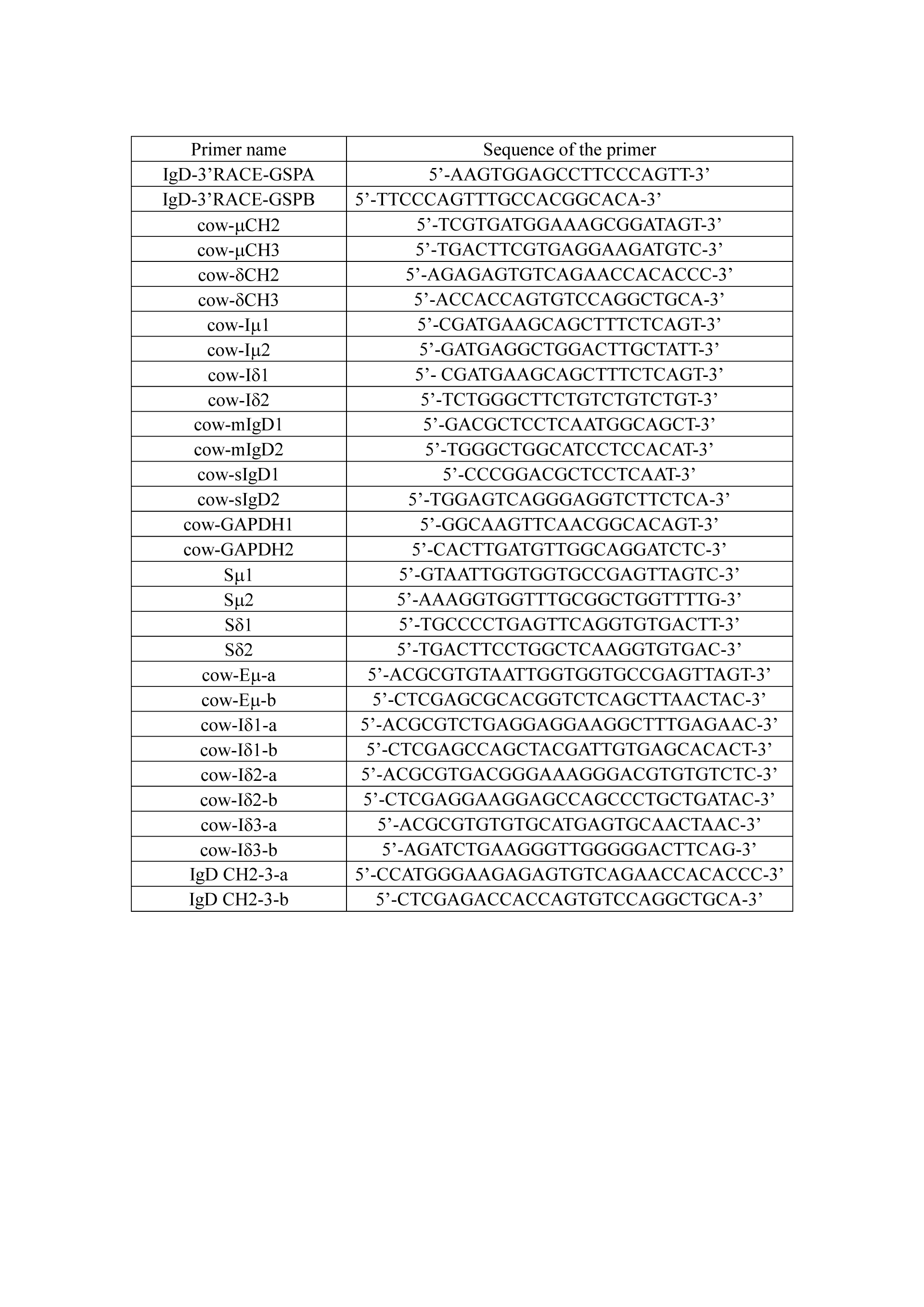

Supplement: Table S1 — Primers used in this study. (TIF) [file pone.0044719.s001.tif]

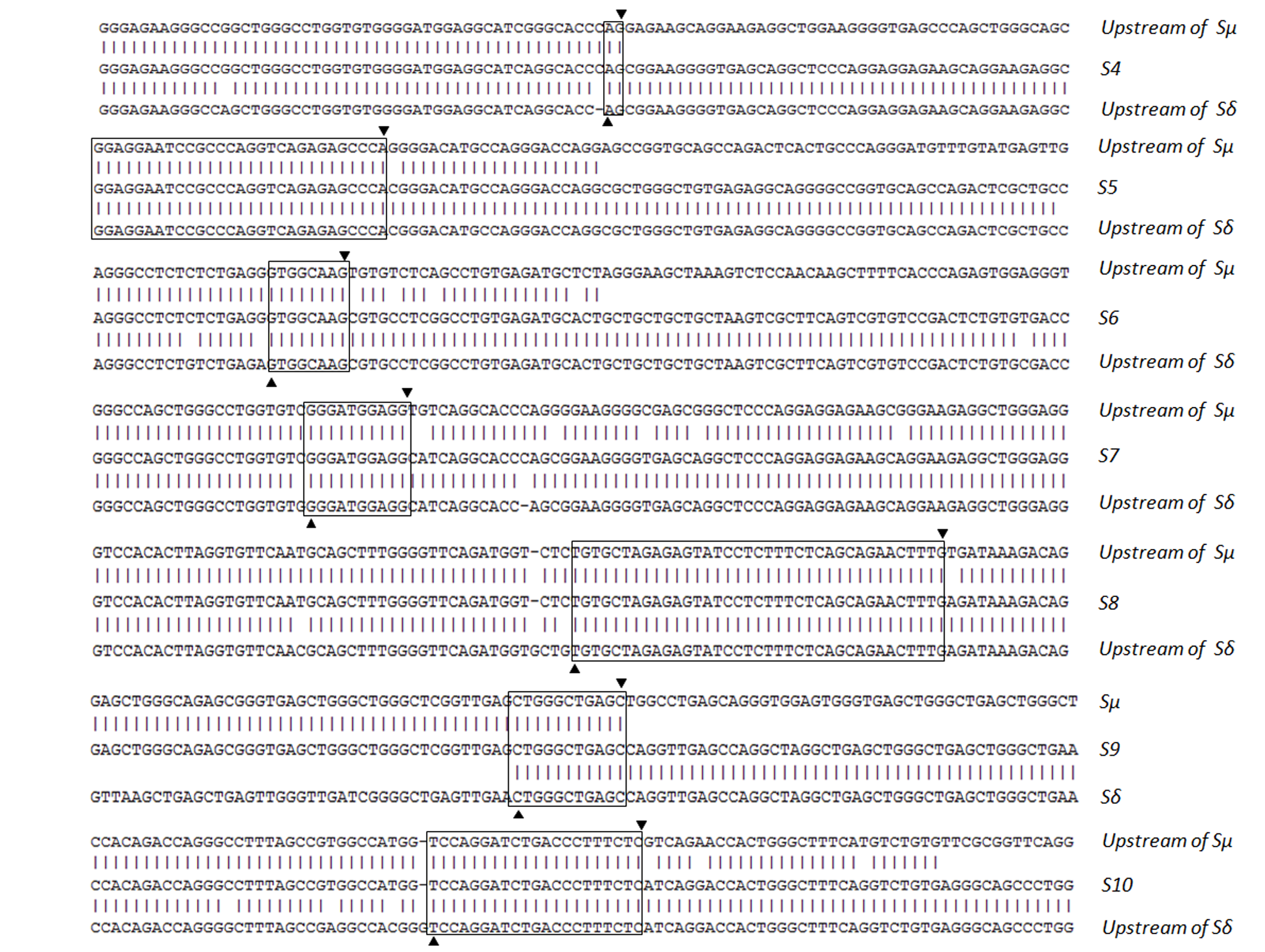

Supplement: Figure S1 — DNA sequence of the recombined Sμ-Sδ junctions. Upper sequence, bovine germline Sμ region (accession no. AY158087); lower sequence, bovine germline Sδ region (accession no. AF411241). The middle sequences are the cloned PCR products. Identical nucleotides are shown by vertical lines. The Sμ and Sδ breakpoints are represented by inverted triangles and forward triangles, respectively. Overlaps are indicated by boxes. (TIF) [file pone.0044719.s002.tif]

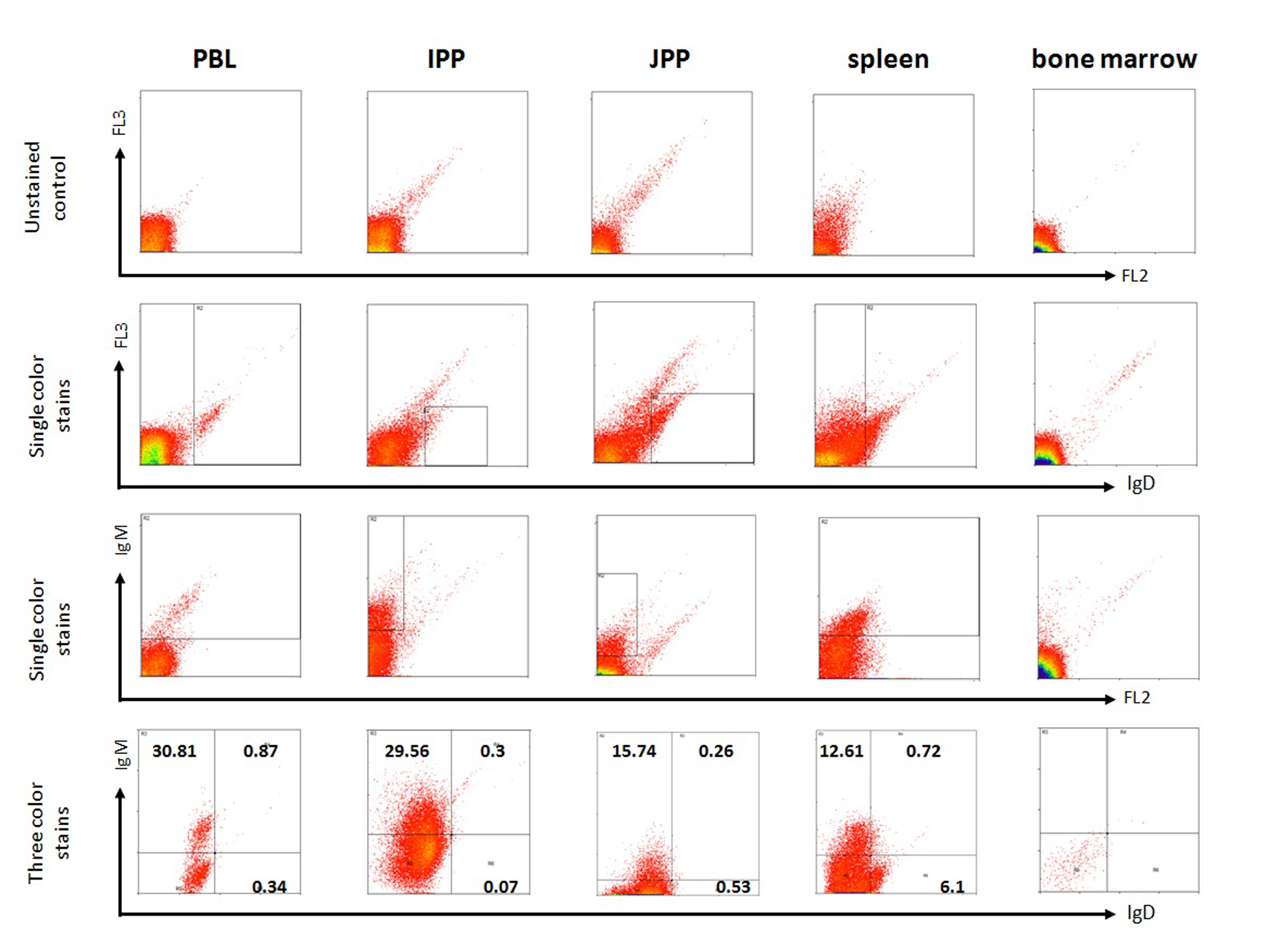

Supplement: Figure S2 — Control plots for the flow cytometric analysis. FACS dot plots show the fluorescence of unstained cells from the PBLs, IPP, JPP, spleen and bone marrow separately (top panel) and the fluorescence area of cells stained with either PE-conjugated anti-bovine IgD (second panel) or biotin-labeled anti-bovine IgM plus PE/Cy5-labeled streptavidin (third panel) alone. The bottom panel shows the result of FACS using FITC-conjugated anti-bovine B220, PE-conjugated anti-bovine IgD and biotin-labeled anti-bovine IgM plus PE/Cy5-labeled streptavidin together. (TIF) [file pone.0044719.s003.tif]
